# Sera from Visceral Leishmaniasis Patients Display Oxidative Activity and Affect the TNF-*α* Production by Macrophages* In Vitro*

**DOI:** 10.1155/2017/5861453

**Published:** 2017-11-05

**Authors:** Neci M. Soares, Joelma N. de Souza, Tatiana F. Leal, Eliana A. G. Reis, Maria S. Miranda, Washington L. C. dos Santos, Márcia C. A. Teixeira

**Affiliations:** ^1^Departamento de Análises Clínicas e Toxicológicas, Faculdade de Farmácia, Universidade Federal da Bahia, Salvador, BA, Brazil; ^2^Centro de Pesquisa Gonçalo Moniz, Fundação Oswaldo Cruz, Salvador, BA, Brazil

## Abstract

Mammalian protection against leishmanial infection depends on the development of an effective immune response. Zoonotic visceral leishmaniasis (ZVL) patients are usually unable to mount an effective immune response against the parasite and indeed appear to be severely immunosuppressed. This suppression has strong nonspecific and specific components mediated by serum factors and leishmanicidal activity of infected macrophages, respectively. The lipid profile has been shown to be altered in ZVL patients' sera. This work aimed at (i) determining the HDL, Apo A1, LDL, and VLDL concentrations in ZVL patients' sera; (ii) investigating the oxidative effect of ZVL patients' sera on the *β*-carotene matrix; (iii) measuring IL-10, IL-6, IL-12p40, and tumour necrosis factor-*α* (TNF-*α*) concentrations in the macrophage cultures, to which 10% of ZVL patients' serum had been added. Levels of HDL, LDL fraction, and apolipoprotein A1 in ZVL patients' sera were lower than those of healthy individuals' sera, except for the mean level of VLDL. The matrix of *β*-carotene and linoleic acid system was oxidized in the presence of ZLV patients' sera. The presence of ZVL patients' sera did not modify the cytokine production of IL-6, IL-12p40, and IL-10 by human macrophages* in vitro* but TNF-*α* production was altered, probably due to lack of macrophage stimulation by lipoprotein.

## 1. Introduction

Zoonotic visceral leishmaniasis (ZVL), caused by the intracellular protozoan parasite* Leishmania infantum*, is a tropical disease that is often fatal when untreated [1]. ZVL patients are usually unable to mount an effective immune response against the parasite and indeed appear to be immunosuppressed. This suppression, which is the major cause of death, has a strong nonspecific component, which may be mediated by serum factors [2, 3].

For instance, the lipid profile has been found to be low in ZVL patients' sera. Low LDL concentration found in the sera of many visceral leishmaniasis patients affects the* in vitro *production of cytokines by macrophages. The values of HDL and apolipoproteina A (Apo A) are about 6 and 2.4 times lower in ZVL than in normal individuals' sera, respectively [4]. The low level of HDL may lead to a low paraoxonase 1 (PON1) activity. PON1 is an esterase and lactonase that confer resistance to experimental infection with* T. brucei brucei *and to* Nippostrongylus brasiliensis *and is found in the circulation bound to HDL [5]. Apo A1, which is the major structural and functional protein component of HDL, is necessary for stabilizing and maintaining optimal PON1 activity [6]. HDL also prevents the formation of oxidized LDL, inactivates oxidized phospholipids, inhibits monocyte-to-macrophage differentiation, and has anti-inflammatory effects on macrophage through inhibition of CD11 activation [7–9].

Individuals with asymptomatic or self-healing oligosymptomatic* L. infantum *infection, as well as other asymptomatic individuals who eventually develop progressive ZVL, respond* in vitro* to* Leishmania *antigen with the production of IFN-*γ* and IL-2 [2]. When the disease is already established, however, increased levels of the proinflammatory cytokines IFN-*γ*, tumour necrosis factor-*α* (TNF-*α*), IL-6, and IL-8 can be found in visceral leishmaniasis (VL) patients' sera [10–13]. Despite the increase in IFN-*γ* and TNF-*α* sera levels during active disease, the number of TNF-*α*-containing monocytes and the production of IFN-*γ* by antigen-stimulated peripheral blood mononuclear cells (PBMC) are reduced when compared to asymptomatic and cured individuals [11, 13]. IL-10, which is a potent immunosuppressive and anti-inflammatory cytokine, is expressed in individuals with active VL and decreases after resolution of the disease [11, 13–16]. An important role of IL-10 in* Leishmania *infection may be its ability to block the IFN-*γ* mediated activation of macrophages to kill intracellular protozoa, to inhibit host resistance, and to promote disease-associated pathology [11, 13, 17].

In this work, we compared the oxidant activity of ZLV patients' sera with that of normal individuals' sera. We also compared the pattern of cytokines produced* in vitro* by human macrophages in the presence of ZLV patients' or normal individuals' sera.

## 2. Methods

### 2.1. Sera Samples

A total of 15 blood samples, for serum preparation, were collected before chemotherapy, from 15 parasitologically diagnosed patients with ZVL, aged over 18 and seen at the Endemic Diseases Unit, Pirajá da Silva in Jequié (PIEJ), Bahia, Brazil. Control sera were collected from 15 age- and sex-matched (seemingly healthy) of same endemic region individuals, who did not have detectable anti-*Leishmania* antibody by ELISA [18]. All patients and volunteers were previously informed of the nature of the research and agreed to participate in the study. The project was approved by the Committee of Ethics in Research of the Gonçalo Moniz Research, Oswaldo Cruz Foundation.

### 2.2. Evaluation of Oxidant Activity of the ZVL Patients' Sera Using the *β*-Carotene and Linoleic Acid System

Oxidant activity was assessed according to the methodology described by Marco (1968) [19] and modified by Fernández-Miranda et al. (1998) [20]. Fifteen ZVL patients' and 15 healthy individuals' sera were tested. The *β*-carotene solution was prepared by dissolving 1 mg of *β*-carotene in chloroform in a round-bottom flask containing 20 mg of linoleic acid and 200 mg of the emulsifier Tween 20 (*β*-carotene system). After removal of the chloroform by rotary evaporator at 50°C, 50 mL of distilled water was added under vigorous stirring. Aliquots (5 ml) of this emulsion were transferred to a series of test tubes containing in duplicate 0.2 mL of sera and initial absorbance (Abs) was read at 470 nm (time zero) in a spectrophotometer (BIOSPECTRO SP-220). The samples were then incubated in a water bath at 50°C for 120 min and the absorbances were read again at 470 nm. The oxidant activity was calculated considering 100% of absorbance in *β*-carotene system without serum (control) using the following formula:

(1) $$\begin{array}{c} \frac{\text{Abs initial of sample}-\text{Abs final of sample}}{\text{Abs initial of control}-\text{Abs final of control}}\times 100. \end{array}$$

### 2.3. Quantification of Sera Lipids

The serum levels of triglycerides, cholesterol and fraction, and apolipoprotein A1 were determined with the use of commercially available kits (Labtest Diagnostica S.A., Belo Horizonte, Brazil), using enzymatic methodologies or Trinder's final reaction (quinone imine formation).

### 2.4. Cell Culture

Healthy donors' PBMC were separated using Ficoll-Paque (Sigma Chemical Co., St. Louis, USA) and cultured in 24-well plates in RPMI medium supplemented with amino acids and 10% normal AB human serum (complete RPMI), at 37°C and 5% CO_2_. After 48 hours of culture, the wells were washed with RPMI at 37°C for removal of nonadherent cells and incubated in complete RPMI medium. On the seventh day 100 *μ*l (10% v/v) of ZLV patients' or healthy individuals' sera was added to wells and the cultures were incubated for 24 hours at 37°C and 5% CO_2_. The culture supernatants were then collected and stored at −20°C.

### 2.5. Quantification of Cytokines

Commercially available detection kits were used to determine the concentrations of IL-6, IL-12p40, TNF-*α* (Duo-set-Kit;* R&D Systems, Inc.*), and IL-10 (Human ELISA Set kit,* BD Biosciences*), following the manufacturers' instructions.

### 2.6. Statistical Analyses

Analyses of the statistical significance of differences in lipid fractions and cytokines production found in ZVL patients and in normal individuals' sera were carried out using the unpaired Student's *t*-test. The correlation between oxidant activity of the ZLV patients' sera and concentrations of HDL fraction was determined by Spearman test. Differences were considered as statistically significant when *p* < 0.05.

## 3. Results

Low levels of HDL, LDL fraction, and apolipoprotein A1 were found in ZVL patients' sera. The mean level of HDL in those sera was 4.4 times lower than that of healthy individual sera. Conversely, the mean level of VLDL was higher in ZVL patients' sera than that found in healthy individuals' sera (*p* < 0.05, Table 1).

The matrix of *β*-carotene and linoleic acid system was oxidized to a higher degree in the presence of ZVL patients' sera than in the presence of healthy individuals' sera (*p* < 0.05). The percentage of oxidation had a negative correlation with the serum HDL-cholesterol concentration (*r* = −0.86, *p* < 0.05, Table 2).

**Figure 1:**
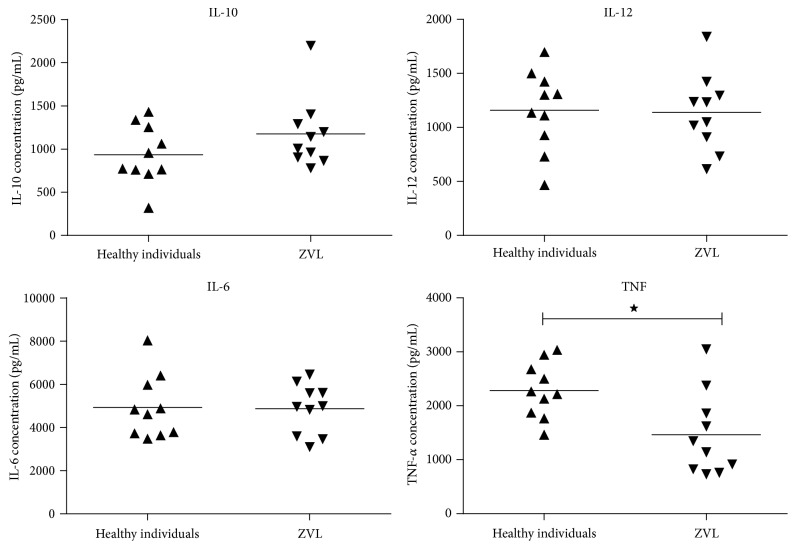
Culture supernatants of PBMC-derived macrophages of healthy individuals were collected 24 hours after the addition of 10% of zoonotic visceral leishmaniasis (ZVL) or healthy individuals' serum to measure cytokine concentrations by ELISA. The data represent the mean of three experiments with cells from different donors. All cultures were done in the presence of 10% normal AB serum. ^★^*p* < 0.05; unpaired Student's* t*-test.

Cytokine production of IL-6, IL-12p40, and IL-10 by PBMC-derived macrophages in the presence of ZLV patients' sera was not altered when compared with cultures in the same conditions in the presence of healthy individuals' sera (*p* > 0.05); however, TNF-*α* production was reduced in the presence of ZLV patients' sera more than in the normal sera (*p* < 0.05, Figure 1).

## 4. Discussion

Lipid disorders have been described in patients with active ZVL [4]. The serum lipid profile of these patients is characterized by hypertriglyceridemia with reduced levels of HDL and apolipoprotein A1. In the present work, it is shown that concentrations of HDL and its major apolipoprotein, Apo A1, and LDL were markedly reduced in ZVL sera when compared with those concentrations in the sera of normal individuals living in the same area. These results are in agreement with Bekaert and collaborators (1989) [21], who reported decreased sera levels of HDL and Apo A1 in 17 Tunisian patients with ZVL, and with Liberopoulos and collaborators (2002) [22], who reported a case of a man with ZVL and severely decreased HDL-cholesterol serum levels. Soares and collaborators (2010) [4] described values of HDL in ZVL patients' sera approximately six times lower than in normal individuals' sera, as well as low values of Apo A1 and LDL. The mechanisms controlling HDL production are poorly understood, and its acquisition by tissues is dependent upon the activity of enzymes such as the endothelial lipoprotein lipase, which may be impaired in patients with VL [23]. HDL has been shown to possess antiapoptotic and anti-infectious functions and its highest antioxidant and anti-inflammatory activities are mainly attributed to Apo A1 and paraoxonase 1 [24, 25]. Various studies have shown that Apo A1 and paraoxonase 1 play a role in antioxidant defense mechanisms avoiding cell damage and development of chronic diseases [26]. It is possible that low concentrations of HDL have led to oxidation of the *β*-carotene matrix in this study. Epidemiological studies have indicated that a negative correlation exists between serum HDL-cholesterol levels and inflammatory chronic diseases [27].

This study presents evidence that the *β*-carotene system is oxidized in the presence of ZVL patients' sera. There is an amount of LDL oxidized in VL patients' sera which is absorbed by the scavenger receptor of macrophages and facilitates foam cell formation, stimulating inflammatory processes [28, 29]. Although ox-LDL concentration was not measured here, even in low concentration, this could oxidize the *β*-carotene system. This may be due to a low concentration of HDL and APO A1 which has the function of protecting LDL from oxidation since the mean level of HDL ZVL patients' sera is 4.4 times lower than that of healthy individuals' sera (*p* < 0.05). Probably the inverse correlation between HDL concentration and matrix oxidation observed here is due to the low HDL concentration.

An imbalance in TNF-*α* production occurs in the course of severe forms of ZVL, and this imbalance has been associated with disease progression and lymphoid tissue disorganization [4, 12, 30]. Physiological concentrations of HDL have an inhibitory effect on the production of TNF-*α* and of IL-1 by endothelial cells and prevent monocyte adhesion to the endothelium [31]. Additionally, a reduced synthesis of apolipoprotein A in the liver and intestine, and an enhanced activity of the LDL receptor, may be triggered by proinflammatory cytokines, such as IL-6 [22, 32]. Soares et al. (2010) [4] showed that HDL limits the production of TNF-*α* by* Leishmania* infected macrophage, so that the low HDL levels below physiological concentrations found in some ZVL patients' sera perhaps contribute to production of proinflammatory cytokines.

The system* in vitro* used here did not show differences in the IL-6, IL-12p40, and IL-10 production by macrophages of healthy individuals in presence of ZVL patients' sera or normal human sera. However, TNF-*α* production by macrophage in the presence of ZVL patients' sera was inhibited, perhaps by the low concentration of LDL, two times lower than those found in normal individuals' sera. A minimal concentration of LDL could be necessary to stimulate the TNF-*α* production by the macrophage infected by* Leishmania in vitro*. We have previously shown that LDL stimulates the production of TNF-*α* by infected PBMC-derived macrophage, and not by uninfected macrophage [4]. The synergism between infection and LDL was obligatory, and neither of them alone led to the production of high concentration of the cytokines [4]. On the other hand, in some reports, the* in vitro* infection of macrophages by* Leishmania* was shown to promote the synthesis of IL-10, IL-6, TNF-*α*, and/or their correspondent mRNA, even in the presence of subphysiological concentrations of sera lipoproteins, that is, the concentrations present in a medium containing 10% normal serum [33–37].

Evidence has provided herein that ZVL patients' sera oxidize the matrix of *β*-carotene and linoleic acid system but do not modify the production of IL-6, IL-12p40, and IL-10 by human macrophages* in vitro*. The TNF-*α* production in the presence of ZVL patients' sera was inhibited, probably due to the low concentration of LDL in ZVL patients' sera.

## Figures and Tables

**Table 1:** Lipid fractions of 15 zoonotic visceral leishmaniasis (ZVL) and 15 healthy individuals' sera.

Lipid fractions and oxidant activity	Concentrations of lipid fractions		Statistical significance^a^
	ZVL patients	Healthy individuals	
VLDL	56 ± 18	27 ± 8	<0.05
LDL	60 ± 19	123 ± 29	<0.05
HDL	9.3 ± 4.1	40 ± 9.3	<0.05
APO A1	101.5 ± 21	198.4 ± 17.5	<0.05

^a^Unpaired *t*-test.

**Table 2:** Oxidant activity of 15 zoonotic visceral leishmaniasis (ZVL) sera and 15 healthy individuals' sera on the *β*-carotene and linoleic acid and linoleic acid system.

Number of individuals	Oxidant activity (%)		Concentrations of HDL fraction (mg/dL)^a^	
	ZLV patients	Healthy individuals	LV patients	Healthy individuals
1	93	32	7	37
2	52	33	5	36
3	86	24	7	39
4	85	23	6	41
5	26	36	18	32
6	52	30	8	34
7	11	51	16	48
8	41	32	9	59
9	25	36	15	32
10	27	32	10	37
11	51	21	7	53
12	94	29	6	42
13	30	48	9	58
14	92	34	5	35
15	25	42	11	31

Mean^b^	52,7 ± 29.7	33,5 ± 8.4	9,3 ± 4.1	40,9 ± 9.3

Correlation between oxidant activity of the ZLV patients' sera and ^a^concentrations of HDL fraction as determined by Spearman test, *r* = −0.86, *p* < 0.05. ^b^Mean concentration in mg·dL^−1^ ± standard deviation of the mean.

## Abbreviations

ZVL: Zoonotic visceral leishmaniasis
PBMC: Peripheral blood mononuclear cells
VLDL: Very low density lipoproteins
LDL: Low density lipoproteins
HDL: High density lipoprotein.
